# Structural validity of the Eating Disorder Examination—Questionnaire: A systematic review

**DOI:** 10.1002/eat.23721

**Published:** 2022-05-03

**Authors:** Paul E. Jenkins, Renee D. Rienecke

**Affiliations:** ^1^ School of Psychology and Clinical Language Sciences University of Reading Reading UK; ^2^ Eating Recovery Center/Pathlight Mood & Anxiety Centers Chicago Illinois USA; ^3^ Department of Psychiatry and Behavioral Sciences Northwestern University Chicago Illinois USA

**Keywords:** assessment, eating disorder, Eating Disorder Examination‐Questionnaire, factor analysis, patient‐reported outcome measures, psychometric

## Abstract

**Objective:**

The main aim was to perform a systematic literature review of studies investigating the factor structure of the Eating Disorder Examination‐Questionnaire (EDE‐Q), a widely used measure of eating pathology. Secondary aims were to summarize the quality of reporting of latent variable (factor) analyses in these studies and review support for different factor solutions.

**Method:**

Literature was identified through Scopus, Medline, PsycInfo, and ProQuest databases published up to February 23, 2022 and outreach via an international listserv. All studies published in English reporting factor analysis of the EDE‐Q were included with few restrictions. Sixty studies including 63,389 participants met inclusion criteria.

**Results:**

The originally proposed four‐factor solution received little empirical support, although few alternative models have been robustly evaluated. Items assessing shape and weight concerns frequently coalesce in factor solutions, suggesting that these constructs are closely related. Investigations of brief versions of the EDE‐Q have produced more consistent findings, suggesting that these measures, particularly a seven‐item version, might be useful alternatives to the full version. Quality of studies was reasonable, with important methodological elements of factor analysis often reported.

**Discussion:**

The findings are of relevance to practitioners and researchers, suggesting that the “original” factor structure of the EDE‐Q should be reconsidered and that use of a seven‐item version is to be encouraged.

**Public Significance:**

Self‐report questionnaires are widely used in the assessment of disordered eating. The current study found that there is little consensus about the structure of a common measure of eating psychopathology. There is more consistent support for a brief, seven‐item, version assessing dietary restraint, body dissatisfaction, and overvaluation of weight and shape.

## Resumen

**Objetivo:**

El objetivo principal fue realizar una revisión sistemática de la literatura de los estudios que investigan la estructura factorial del Cuestionario de Eating Disorders Examination (EDE‐Q), una medida ampliamente utilizada en la patología alimentaria. Los objetivos secundarios fueron resumir la calidad del informe de los análisis de variables latentes (factores) en estos estudios y revisar el apoyo a diferentes soluciones factoriales.

**Método:**

La literatura se identificó a través de las bases de datos Scopus, Medline, PsycInfo y ProQuest publicadas hasta el 23 de febrero de 2022 y de divulgación a través de un servidor de listas internacional. Todos los estudios publicados en inglés que reportaron el análisis factorial de la EDE‐Q se incluyeron con pocas restricciones. Sesenta estudios con 63,389 participantes cumplieron los criterios de inclusión.

**Resultados:**

La solución de cuatro factores propuesta originalmente recibió poco apoyo empírico, aunque pocos modelos alternativos han sido evaluados sólidamente. Los elementos que evalúan las preocupaciones de peso y figura corporal con frecuencia se unen en soluciones factoriales, lo que sugiere que estos constructos están estrechamente relacionados. Las investigaciones de versiones breves del EDE‐Q han producido conclusiones más consistentes, lo que sugiere que estas mediciones, en particular una versión de siete ítems, podrían ser alternativas útiles a la versión completa. La calidad de los estudios fue razonable, y a menudo se reportaron elementos metodológicos importantes del análisis factorial.

**Discusión:**

Los hallazgos son relevantes para los clínicos e investigadores, lo que sugiere que la estructura factorial “original” del EDE‐Q debe reconsiderarse y que se debe alentar el uso de una versión de siete ítems.

## 1 INTRODUCTION

Efficient assessment of eating pathology is integral to experimental studies, epidemiological work and clinical settings, and several psychometric measures have been designed for this need. Whilst EDs have traditionally been considered as discrete “categories” (e.g., American Psychiatric Association, 2013), continuous measures can capture the full variation in eating pathology that is seen in both clinical and non‐clinical samples (e.g., Luo et al., 2016). Such an approach is consistent with a “network perspective” to conceptualizing mental health problems, whereby EDs, as with other mental health problems, are seen as occurring on a spectrum and demonstrate patterns of interacting symptoms with multifactorial causes, rather than existing as discrete disease entities (e.g., Borsboom, 2017; Monteleone & Cascino, 2021).

A number of self‐report measures are widely used in the assessment of eating pathology, one of the most popular of which is the Eating Disorder Examination‐Questionnaire (EDE‐Q; Fairburn & Beglin, 1994, 2008). In line with the semi‐structured interview from which it was derived (the Eating Disorder Examination, or EDE; Cooper & Fairburn, 1987; Fairburn et al., 2008), the EDE‐Q assesses a variety of behaviors and cognitive features relevant to eating pathology, the latter of which are summarized by four subscale scores ([Dietary] Restraint, Eating Concern, Shape Concern, and Weight Concern; Fairburn & Beglin, 2008) obtained from item scores. From a psychometric point of view, although there is support for the reliability and validity of the EDE‐Q in the assessment of ED symptoms (Berg et al., 2012), the suggested factor structure of the measure has proven difficult to corroborate (Grilo et al., 2013), perhaps as the items and constructs of both measures were developed based on “rational rather than empirical grounds” (Cooper et al., 1989, p. 809).

The EDE‐Q includes definitions and time frames for key symptoms and typically takes a few minutes to complete. Twenty‐two “attitudinal” items are scored on a 0–6 scale based on either: (a) number of days in the previous 28; or (b) “Not at all” to “Markedly.” They include questions such as “Have you had a definite fear that you might gain weight?” and “How dissatisfied have you been with your shape?.” Six further “behavioral” items assess the frequency of disordered eating behaviors, such as binge eating and self‐induced vomiting, and are scored on a ratio scale. These items are typically excluded from calculations of subscales, although some authors have included them due to their centrality in the diagnosis of EDs, often by adapting them to a Likert (ordinal) scale (e.g., Hrabosky et al., 2008; Lev‐Ari et al., 2021).

Critiques of these measures have included a bias towards the assessment of bulimia nervosa (Thomas et al., 2014), with a similar criticism that ED measures in general have often been developed with young‐adult females in mind (Forbush et al., 2013; Mitchison & Mond, 2015). Concepts integral to EDs, such as weight and shape concerns, can be problematic to assess and are often difficult for respondents to understand, even when prompted (Thomas et al., 2014). The EDE‐Q aligns with popular cognitive‐behavioral models of eating pathology, presenting items and scoring that reflect the theory that a drive for thinness underpins much eating pathology. However, such an assumption may not hold for those from non‐Western cultures (e.g., Mitsui et al., 2017) or male samples, who typically report lower scores (Schaefer et al., 2018). For example, a study of 1150 adult men suggested that, whilst the concept of body image was relevant, a focus on the “thinness ideal” is restrictive and undervalues the role of muscularity concerns (Forrest et al., 2019). Such difficulties can affect the interpretation of scores as well as affecting the computation of scales assumed to reflect single constructs (e.g., Weight Concern or Shape Concern), with an additional risk that the strength of association between certain items and latent factors (e.g., factor loadings) varies across groups (e.g., Serier et al., 2018; cf. Machado et al., 2018).

As noted above, the attitudinal items of the EDE‐Q can be used to compute four subscales although Eating Concern was not included as a distinct subscale in the original description of the EDE‐Q (Fairburn & Beglin, 1994). A Global score can also be computed by summing the scores of the four subscales and dividing the resulting total by the number of scales (i.e., four) (Fairburn et al., 2008). Widely used in research, the EDE‐Q is also recommended as an outcome measure within the United Kingdom National Health Service (National Collaborating Centre for Mental Health, 2019). However, a lack of support for the structural validity of the subscales of the EDE‐Q can lead to inconsistency around what outcomes are reported, with many studies and clinical services reporting outcomes according to the originally proposed subscales (e.g., for population norms [Hilbert et al., 2012] and treatment studies [e.g., Fischer et al., 2014]). Further, the discriminant validity of the EDE‐Q has been limited by variable item loadings and inconsistent identification with a latent factor (Forbush et al., 2013), and a significant proportion of individuals with anorexia nervosa report Global EDE scores in the “normative” range at pre‐treatment (Thomas et al., 2014).

Given the frequent reliance on self‐report measures in evaluating outcome from treatment and assessing symptoms, clarification of the constructs being assessed, and accurate measurement thereof, is vital (Flake & Fried, 2021; Mokkink et al., 2018 and Prinsen et al., 2018). Internal structure is directly related to scoring and interpretation (Messick, 1995) and the absence of structural validity might undermine support for the (construct) validity of a measure (Keith & Kranzler, 1999). Whilst the clinical utility of the EDE‐Q has often been promoted as a strength, this is likely to be more reliable if a consistent factor structure of the measure can be established.

Investigations of the underlying factor structure (and hence the EDE‐Q's structural validity) have produced inconsistent findings and there has been little systematic evaluation of data‐driven models. Rand‐Giovannetti et al. (2020) evaluated alternative models of the EDE‐Q factor structure in a sample of 940 undergraduate students. They concluded that a four‐factor model (without a higher order factor representing the “Global” score and with some differences to the “Original” model) provided the best fit, although fit statistics were similar across several competing models (Sellbom & Tellegen, 2019). To define models for their study, they identified 24 studies looking at the factor structure of the EDE‐Q (generating almost as many unique latent structures). Aside from a four‐factor model of attitudinal items (often labeled the “Original” model), alternative factor solutions have combined items from two factors (“Shape Concern” and “Weight Concern”; e.g., Peterson et al., 2007), provided different interpretations of the full scale (e.g., Becker et al., 2010; Friborg et al., 2013), or reduced the number of items by removing those which do not consistently load onto a factor (e.g., Gideon et al., 2016; Grilo et al., 2015; Hrabosky et al., 2008). In many studies, a novel interpretation of latent dimensions is presented, oftentimes departing only slightly from existing suggestions. Whilst sample differences, for example, might explain heterogeneity in findings regarding the factor structure of the EDE‐Q, it is also possible that methodological differences, such as how factor analysis was performed, may account for discrepancies in proposed solutions (e.g., Vogt et al., 2017), an issue not considered in depth by Rand‐Giovannetti et al. Methods to determine model fit, for example, can be susceptible to multivariate non‐normality (Fabrigar et al., 1999) and processes for establishing how many factors to retain in exploratory factor analysis (EFA) are often debated (Preacher et al., 2013).

Although the summary provided by Rand‐Giovannetti et al. (2020) is helpful, there have been more than 25 investigations of the EDE‐Q's factor structure since the initial online publication of this paper, and so a systematic review of all existing literature is warranted. Further, it is unclear how systematically and comprehensively the literature was reviewed—given that this was not the primary aim of their study—and some previous studies that may be relevant (e.g., Machado et al., 2014) do not appear to have been included. A review of the EDE‐Q's structural validity, which is the primary aim of the current study, would help focus efforts to refine use of the EDE‐Q and to suggest where the weight of evidence lies regarding an optimal factor structure and recommendations for its use in both clinical and research settings. A further goal of the current study is to formulate recommendations for the conduct of future studies, as has been done in other areas (e.g., DiStefano & Hess, 2005; Jackson et al., 2009). Whilst establishing the clearest factor structure of a measure is only part of an evaluation of its utility, this is necessary for the appropriate assessment of internal consistency (Mokkink et al., 2018) as well as for tests of measurement invariance, which afford (mean) comparison across different groups.

## 2 METHODS

### 2.1 Protocol and registration

Studies were eligible if they provided full‐texts in English and included latent variable analysis (EFA or confirmatory factor analysis, CFA) of the EDE‐Q. All versions of the EDE‐Q (i.e., where all or some of the items from the measure are included) were considered for inclusion in the review, although youth and child versions were not included as these were developed based on “major changes” (Goldschmidt et al., 2007, p. 462) to the EDE‐Q, which, alongside possible developmental differences (Forsén Mantilla et al., 2017), might affect psychometric properties. Searches were conducted from 1993 (just before the EDE‐Q was first published) to February 23, 2022. The protocol was registered on PROSPERO and can be accessed at https://www.crd.york.ac.uk/prospero/display_record.php?RecordID=245357 and the review followed the Preferred Reporting Items for Systematic reviews and Meta‐Analyses (PRISMA) guidelines (Page et al., 2021; Table S1).

### 2.2 Search strategy

Three electronic databases were searched (Scopus, Medline, PsycInfo) using the following search terms in All Fields: “EDE‐Q" AND “factor analysis” OR “exploratory factor analysis” OR “confirmatory factor analysis” OR “factor structure.” A search of gray literature was also conducted by using the same search terms through ProQuest and posting a request for relevant literature on an international eating disorders listserv. Where Abstracts indicated that latent variable analysis (LVA) of the EDE‐Q was conducted, full texts were subsequently reviewed. Following the database search, reference lists of identified studies were searched for additional studies to be included in the review. Abstracts were collated into an electronic document and duplicates were removed.

### 2.3 Selection criteria

There were no restrictions on the population covered (e.g., gender and participant nationality). Studies were only included if they described an investigation of the structural validity of the EDE‐Q (including some or all of its original items), either as a primary objective or as part of a wider investigation of its psychometric properties. When the EDE‐Q was translated into another language, this was included as long as the previous criterion was met. Similarly, studies using multi‐group CFA (e.g., testing for measurement invariance) were included if an analysis of factor structure was reported, and only findings relating to structural validity are discussed in this review.

The current review collated studies of the structural validity of the EDE‐Q. Assessments solely of unidimensionality (i.e., the structural validity of a single subscale) were not considered. Key information about EDE‐Q studies using factor analysis was summarized and findings organized to inform recommendations for the most appropriate subscales to report. The review also aimed to summarize the factor solution(s) with the most consistent evidence.

### 2.4 Data extraction

Around 80% of abstracts were assessed by both authors, suggesting good agreement (κ = .86). The authors independently selected full texts for inclusion in the review (Li et al., 2021), noting the: type of analysis used (e.g., EFA and CFA); population sampled (including country, gender, age, race/ethnicity, and socioeconomic status); sample size; and language used (Tables 1, S2–S5). In the case of EFA, details of the software used, association matrix (e.g., correlation), estimation method, nature of rotation (e.g., varimax and promax), and criteria for factor selection (e.g., scree plot and Kaiser–Guttman criterion) were recorded (e.g., Henson & Roberts, 2006). Presence of a pattern matrix and reference to communalities were also noted. For CFA studies, the estimation procedure (e.g., maximum likelihood), software used, matrix analyzed, and whether more than one model was tested were recorded (e.g., Jackson et al., 2009). Whether studies made relevant statements about missing data, normality, and fit indices (including whether cutoffs were reported a priori) was also noted. The strategy was piloted on three papers to refine the extraction template and to ensure consistency across reviewers, following which independent reviews of the remaining 75 papers were performed.

**TABLE 1:** Sample characteristics of studies included in current review

Secondary school/university students								
First author, year	N	Setting, population	Country/EDE‐Q language	Gender	Mean age (range)	Mean BMI (range)	Race/ethnicity^a^	Socioeconomic status
Asl, 2021 (CFA)	302	University students	Iran/Persian	44% female	23.83 (18–46)	NR	NR	NR
Baceviciene, 2020 (CFA)	382	Undergraduate and graduate students	Lithuania/Lithuanian	75.1% female	24 (NR)	NR (15.8–36.2)	NR	NR
Becker, 2010 (EFA)	523	Secondary school students	Fiji/Fijian or English	100% female	16.67 (15–20)	23.97 (NR)	Ethnic Fijians: 100%	NR
Chan, 2015 (CFA)	310	University students	Hong Kong/English or Chinese	54.2% female	20.75 (18–26)	Females: 20.05 (13.89–34.80); Males: 20.64 (14.03–32.87)	Asian: 100%	NR
Franko, 2012 (CFA)	173	College students	USA/English	100% female	19.8 (18–22)	23.9 (NR)	Caucasian: 42.7%; Latina: 57.3%	NR
Giovazolias, 2013 (CFA)	500	Undergraduate students	Greece/Greek	100% female	20.55 (17–44)	21.72 (NR)	Greek: 100%	NR
Goel, 2022 (CFA)	685	University students	USA/English	100% male	19.77 (NR)	24.40 (NR)	Asian/Hawaiian/ Pacific Islander: 23.8% Black: 22.6% White: 53.6%	NR
Grilo, 2015 (CFA)	801	University students	USA/English	71.54% female	20 (18–47)	23.5 (NR)	Caucasian: 82.8% Other: NR	NR
He, 2021 (CFA)	1068	Full‐time undergraduate students	China/Chinese	52.6% female	20.11 (17–24)	21.11 (14.30–36.89)	Chinese: 100%	NR
Jenkins, 2020 (CFA)	405	University students	UK/English	60.25% female	20.71 (17–51)	23.14 (14.68–59.12)	NR	NR
McEntee, 2021 (CFA)	1173	Undergraduate students	USA/English	73.6% female	19.41 (18–25)	23.87 (15.94–46.59)	Latinx: 62.3% Non‐Latinx White: 37.7%	NR
Mitsui, 2017 (EFA)	1430	Undergraduate students	Japan/Japanese	81.75% female	19.4 (NR)	20.6 (NR)	Japanese: 100%	NR
Mohd Taib, 2020 (EFA)	94	Undergraduate students	Malaysia/Malay	83% female	22.5 (20–26)	NR (NR)	Malay: 91.5%; Chinese: 1.1%; Other: 7.4%	NR
Mohd Taib, 2021 (EFA)	595	University students	Malaysia/Malay	57% female	21.9 (19–28)	23.2 (14.4–47.7)	Malay: 98.3%; Other: 1.7%	NR
Penelo, 2013 (CFA)	2928	Adolescent students	Mexico/Spanish	52.7% female	15.1 (11–18)	Females: 22.52 (NR) Males: 22.24 (NR)	Mexican: 100%	Hollingshead's index: Females: Low: 32.9%; Medium‐low: 27.8%; Medium: 18.0%; Medium‐high: 14.2%; High: 7.1% Males: Low: 27.6%; Medium‐low: 25.2%; Medium: 21.8%; Medium‐high: 16.9%; High: 8.6%
Ramli, 2008 (EFA)	298	Secondary school students	Malaysia/Malay	52.7% female	NR (12–17)	NR (NR)	Malay: 63.4%; Chinese: 28.2%; Indian and Other: 6.7%	Parents' income: <RM 1000: 25.2%; RM 1001–5000: 39.3%; >RM 5000: 10.1%; Unknown: 25.5%
Rand‐Giovannetti, 2020 (CFA)	940	Undergraduate psychology students	USA/English	69.9% female	20.34 (16–48)	23.28 (NR)	Asian/Pacific Islander: 50.7%; Caucasian: 20.6%; Bi/Multiracial: 16.3%; Native Hawaiian/ Native American/ American Indian: 5.3%; Hispanic: 3.8%; Black: 1.0%; Other: 1.6%	NR
Rica, 2021 (CFA)	796	University students	Spain/Spanish	100% male	19.8 (NR)	22.4 (NR)	Spanish: 100%	NR
Serier, 2018 (CFA)	561	Undergraduate students	USA/English	100% female	20.11 (18–38)	23.76 (NR)	NR	Highest level of education: High school diploma: 49.4%; Working on undergraduate degree: 46.7%
Serier, 2021 (CFA)	150	Undergraduate students	USA/English	100% female	21.29 (18–40)	25.06 (16.31–42.43)	American Indian/Alaska Native: 100%	NR
Villarroel, 2011 (CFA)	708	Undergraduate students	Spain/Spanish	100% female	22.0 (18.3–30.9)	NR (NR)	NR	Hollingshead's index: Low: 20.5%; Medium: 69.9%; High: 9.6%
Zickgraf, 2020 (CFA)	9910	College students participating in Healthy Bodies study	USA/English	54.3% female	23.4 (18–NR)	NR (NR)	Non‐Hispanic White: 66.9%; Non‐Hispanic Black/African American: 5.4%; Hispanic/Latinx: 9.4%; Asian: 13.3%; Other: 5.1%	NR
*Community samples*								
Friborg, 2013 (EFA, CFA)	1076	Representative community sample	Norway/Norwegian	100% female	36.2 (16–50)	24.6 (13.5–55.1)	Norwegian: 100%	Employment status: Working, 72%; Studying, 13.8%; Sick leave, 9.5%; Working at home, 2.5%; Unemployed, 1.7%
Hilbert, 2012 (EFA)	2520	Nationally representative sample	Germany/German	53.7% female	50.5 (14–95)	25.25 (14.17–55.40)	German: 96.7%; Other: 3.3%	Household income <EUR 2000: 56.1%
Kliem, 2016 (CFA)	2508	Nationally representative sample	Germany/German	54% female	49.67 (14–92)	NR (NR)	German: 100%	Household income per month: Phase 1 study: <1250 EUR: 20.4%; 1250 to 2499 EUR: 51.8%; >2500 EUR: 24.7%; Missing: 3.1% Phase 2 study: <1250 EUR: 20.6%; 1250 to 2499 EUR: 45.7%; >2500 EUR: 30.7%; Missing: 3.0%
Lev‐Ari, 2021 (CFA)	1160	Community volunteers	Israel/Hebrew	83.8% female	28.79 (18–76)	23.46 (16.31–53.15)	Israeli: 100%	NR
Melisse, 2021 (EFA, CFA)	2690	Convenience sample from community	Saudi Arabia/Arabic or English	78% female	24.7 (14–81)	31.0 (NR)	Saudi nationals: 97.4%; Other: NR	Highest occupation completed: High school, 27.7%; University in Saudi Arabia, 25.5%; University in another Arab country, 2.1%; University in a Western country, 1%; Employed, 20.4%; Unemployed, 14.9%; Other, 8.1%
Prnjak, 2020 (EFA, CFA)	279	Community sample	Croatia/Croatian	77.1% female	24.61 (NR)	23.18 (NR)	NR	NR
White, 2014 (EFA, CFA)	917	Community sample	UK/English	56.9% female	15.2 (14–18)	NR (NR)	White British: 74.9%; Missing: 15.5%; Other: NR	NR
Zohar, 2017 (EFA, CFA)	292	Community volunteers	Israel/Hebrew	82.5% female	33.39 (19–74)	23.4 (15.4–42.2)	Israeli: 100%	College degree: 59.9%
*Clinical samples*								
Aardoom, 2012 (EFA)	935	Treatment‐seeking patients with EDs	Netherlands/Dutch	100% female	28.97 (12–64)	21.62 (NR)	Caucasian: 92.9%; Other: NR	Education level: General Sample: Low, 16.2%; Intermediate, 26.4%; High, 57.4%; ED Sample: Low, 25.3%; Intermediate, 48.7%; High, 19.6%; Obese Sample: Low, 38.9%; Intermediate, 40.2%; High, 20.9%
Calugi, 2017 (CFA)	264	Inpatients and outpatients with EDs	Italy/Italian	97.3% female	Patients: 22.2 (NR)	Patients: 17.1 (NR)	Italian: 100%	Education level: Patients: Middle school, 39%; High school diploma, 49.2%; Bachelor's degree, 11.7%; Controls: Middle school, 57.4%; High school diploma, 36.1%; Bachelor's degree, 6.5%
Gideon, 2016 (EFA, Rasch)	489	Patients from 3 ED services	UK/English	90.2% female	31.5 (18–72)	AN‐R: 14.23 (NR); AN‐BP: 14.79 (NR); BN: 24.83 (NR); BED: 37.23 (NR); OSFED: 27.27 (NR)	NR	NR
Otani, 2021 (EFA, CFA)	148	Clinical sample of patients with EDs from 5 hospitals	Japan/Japanese	100% female	AN‐R: 29.6 (NR) AN‐BP: 30.9 (NR) BN: 30.7 (NR)	AN‐R: 15.3 (NR) AN‐BP: 15.9 (NR) BN: 22.9 (NR)	Japanese: 100%	NR
Peterson, 2007 (EFA)	203	Females with bulimic symptoms	USA/English	100% female	25.7 (18–57)	23.0 (16.2–53.4)	Caucasian: 90.6%; Asian: 3.4%; Black: 2.5%; Hispanic: 1.5%; Other: 2.0%	Education level: High school or less, 6.9%; Some college education, 64.5%; College degree, 14.3%; Graduate education, 12.3%; Other, 2%
Phillips, 2018 (EFA)	169	Inpatient sample with AN	USA/English	100% female	34.1 (18–69)	15.87 (12.23–18.48)	Caucasian: 94.7%; Other: NR	NR
*Mixed samples*								
Allen, 2011 (CFA)	439	Outpatients with EDs; community participants	Australia/English	100% female	Outpatients: 26.02 (16–72) Community sample: 21.03 (17–50)	AN: 15.81 (NR); BN: 22.31 (NR); EDNOS: 21.18 (NR) Community sample: 21.79 (NR)	ED Sample: Caucasian: 92%; Asian: 2%; Other: 6%; Community Sample: Caucasian: 75%; Asian: 20%; Other: 5%	NR
Barnes, 2012 (CFA)	569	University students; participants recruited from UK‐based ED charities	UK/English	Student sample: 91.8% female; Charity sample: 95.8% female	NR (NR)	NR (NR)	NR	NR
Carey, 2019 (EFA, CFA)	2459	Two student samples; one nonstudent sample	UK/English	Student sample #1: 79.2% female; Student sample #2: 74.9% female; Nonstudent sample: 76.7% female	Student sample #1: 19.89 (17–30) Student sample #2: 22.33 (18–37) Nonstudent sample: 33.08 (18–61)	Student sample #1: 22.69 (NR) Student sample #2: 23.53 (NR) Nonstudent sample: 25.96 (NR)	NR	NR
Carrard, 2015 (CFA)	277	Participants with sub/threshold BED; community sample	Switzerland/French	100% female	Participants with BED: 38.5 (NR) Community sample: 28.1 (NR)	Participants with BED: 31.4 (NR) Community sample: 21.0 (NR)	NR	NR
Compte, 2019 (CFA)	986	Four community samples (college students, weightlifters, cross‐fit gym users, rugby players)	Argentina/Spanish	100% male	College students: 23.53 (18–62) Weightlifters: 29.24 (18–68) Cross‐fit sample: 29.86 (18–59) Rugby players: 21.71 (18–36)	College students: 23.96 (NR) Weightlifters: 25.13 (NR) Cross‐fit sample: 25.37 (NR) Rugby players: 26.86 (NR)	NR	NR
Darcy, 2013 (EFA, CFA)	1634	Competitive athletes; comparison group	USA/English	59.5% female	20.87 (18–26)	23.32 (NR)	Caucasian: 74.2%; Asian: 11.2%; Hispanic: 6.1%; Black: 5.4%; Native Hawaiian/ Pacific Islander: .1%; Biracial or Multiracial: 1.6%; Other: 1.0%	NR
Forbush, 2011 (EFA)	840	Community sample; student sample	USA/English	55% female	Community sample: 38.24 (NR) Student sample: 19.7 (NR)	Community sample males: 26.77 (NR) Community sample females: 25.95 (NR) Student sample males: 24.6 (NR) Student sample females: 22.7 (NR)	Community sample: Caucasian: 89.2%; African‐American: 2.2%; Hispanic/ Latino(a): 2.2%; Asian‐American: 6.4% Native American/ Alaskan Native: .5%; Native Hawaiian/ Pacific Islander: .5% Other: 2.2% Student sample: Caucasian: 91.2%; African‐American: 2.8%; Hispanic/ Latino(a): 3.7%; Asian‐American: 4.4% Native American/ Alaskan Native: .5%; Native Hawaiian/ Pacific Islander: .5% Other: .9%	NR
Lichtenstein, 2021 (CFA)	1331	Clinical sample with EDs (AN, BN, UED); Patients with symptoms of BED; recreational athletes; elite athletes	Denmark/Danish	70.5% female	ED: 27.6 (15–64) BED: 39.1 (18–68) Recreational athletes: 29.6; (15–70) Elite athletes: 20.0 (15–47)	ED: 22.4 (7.6–54.3) BED: 37.8 (16.6–76.2) Recreational athletes: 23.7 (15.2–42.0) Elite athletes: 21.8 (16.6–30.8)	NR	NR
Machado, 2014 (EFA)	554	Clinical sample of patients with EDs; treatment‐seeking sample of patients with obesity	Portugal/Portuguese	100% female	AN: 22.0 (NR) BN: 26.1 (NR) BED: 30.6 (NR) EDNOS: 19.5 (NR) Obese: 41.6 (NR)	AN: 16.5 (NR) BN: 21.4 (NR) BED: 32.2 (NR) EDNOS: 20.55 (NR) Obese: 44.21 (NR)	Portuguese: 100%	NR
Machado, 2018 (CFA)	4726	Student samples; clinical sample of patients with EDs	Portugal/Portuguese	99.64% female	High school students: 16.2 (12–23) College students: 21.7 (17–58) Clinical sample: 23.8 (11–61)	High school students: 20.8 (NR) College students: 22.2 (NR) Clinical sample: 20.4 (NR)	Portuguese: 100%	NR
Machado, 2020 (CFA)	3626	Two clinical samples with EDs; one student sample	Portugal/Portuguese	100% female	Clinical sample #1: 26.18 (13–49) Clinical sample #2: 27.81 (18–49) Student sample: 18.89 (12–58)	Clinical sample #1: 20.57 (NR) Clinical sample #2: 22.68 (NR) Student sample: 21.49 (NR)	NR	NR
Tobin, 2019 (CFA)	1561	Canadian university students and an American crowdsourced community sample	Canada/English	85.2% female	Canadian university females: 20.3 (17–52) Canadian university females and males: 20.5 (18–53) American community sample: 38.6 (19–79)	Canadian university females: 22.3 (14.8–42.9) Canadian university females and males: 22.2 (15.6–37.3) American community sample: 29.3 (15.0–107.9)	Caucasian: 64.0%; Other: NR	NR
Unikel Santoncini, 2018 (CFA)	481‐487^b^	University students; clinical sample of patients with EDs	Mexico/Spanish	100% female	20 (13–47)	22.9 (NR)	Mexican: 100%	NR
Wood, 2016 (EFA)	131	Volunteers treated for ED or self‐identified as someone with eating concerns	USA/English	88.5% female	24.79 (18–56)	26.09 (17–55.5)	Black/African‐American: 14.5%; Hispanic/Latino: 14.5% White/ Caucasian: 54.2%; Native Hawaiian/ Pacific Islander: .0%; Asian: 9.2% Bi/Multiracial: 6.9%; Other: .1%	NR
*Specified non‐clinical samples*								
Grilo, 2013 (CFA)	174	Individuals seeking bariatric surgery	USA/English	75% female	42.9 (NR)	50.2 (NR)	Caucasian: 68%; Black: 17.8%; Hispanic: 7.5%; Other: 6.3%	Some college education: 74%
Heiss, 2018 (CFA)	518	Vegan and omnivore participants	USA/English	74.3% female	26.78 (18‐NR^a^)	23.65 (NR^c^)	White: 73.7%; Other: NR	NR
Heiss, 2020 (CFA)	518	Vegan and omnivore participants	USA/English	74.3% female	26.78 (18–74)	23.65 (12.73–44.28)	White: 73.7%; Other: NR	NR
Hrabosky, 2008 (EFA, CFA)	337	Individuals seeking bariatric surgery	USA/English	83.4% female	43.2 (18–71)	51.4 (33.8–84.9)	White: 69%; Black: 16.5%; Hispanic: 12%; Asian: .5%; Unspecified: 2%	Education level: Completed high school, 91.5%; Some college education, 61.5%
Klimek, 2021 (CFA)	962	Cisgender sexual minority men and women	USA/English	50.2% female	23.68 (18–30)	NR (NR)	White: 38.6%; Black/African‐American: 30.6%; Asian/Pacific Islander: 28.3%; Native American/American Indian: 2.4%; Hispanic/Latinx: 24.3%	NR
Lewis‐Smith, 2021 (EFA, CFA)	1413	Urban adolescents in India	India/English	44.9% female	13 (11–15)	NR (NR)	Born in India: 98.4%; Other: NR	Parents' highest education: Fathers: Primary school, 3.8%; Secondary school, 25.7%; Bachelor's degree, 35.5%; Master's degree, 22.3%; Mothers: Primary school, 4.6%, Secondary school, 23.8%; Bachelor's degree, 35.3%; Master's degree, 24.1%
Parker, 2015 (EFA, CFA)	108	Individuals seeking bariatric surgery	Australia/English	80.6% female	46 (22–70)	33.6 (24.0–50.6)	NR	NR
Parker, 2016 (EFA, CFA)	405	Individuals seeking bariatric surgery	Australia/English	79.3% female	43.8 (20–69)	42.5 (30.2–71.5)	NR	NR
Peterson, 2020 (EFA)	249	Treatment‐seeking transgender youth	USA/English	27.71% TG female	17.04 (11–24)	26.54 (13.46–58.26)	NR	NR
Scharmer, 2020 (CFA)	703	Heterosexual and sexual minority males	USA/English	100% male	33.76 (18–67)	NR (NR)	Caucasian: 74.1%; Other: NR	NR

Abbreviations: AN‐R, Anorexia nervosa, restricting subtype; AN‐BP, Anorexia nervosa; binge eating/purging subtype; BED, Binge eating disorder; BMI, Body mass index; BN, Bulimia nervosa; CFA, Confirmatory factor analysis; ED, Eating disorder; EDE‐Q, Eating Disorder Examination – Questionnaire; EDNOS, Eating disorder not otherwise specified; EFA, Exploratory factor analysis; EUR, Euro; NR, Not reported; OSFED, Other specified feeding or eating disorder; RM, Malaysian Ringget; TG, Transgender; UED, Unspecified eating disorder.

^a^ For consistency, the terms used in this column are taken from the original paper.

^b^ Number varied depending on model.

^c^ This information was not reported in Heiss et al. (2018) but may be the same sample as Heiss et al. (2020).

Following independent selection of full‐texts, the authors compared responses and identified any discrepancies or omissions (e.g., where only one author had recorded a methodological element of the study). Inter‐reviewer agreement for inclusion of studies was good (κ = .85). The full‐texts were re‐read to ensure that the information was, in fact, presented and this was recorded on an electronic database of all studies. If crucial information was unclear, an attempt was made to contact the corresponding author of the study.

### 2.5 Quality assessment and data synthesis

Assessment of the methodological quality of the studies included elements of COSMIN standards (Mokkink et al., 2018) and reporting of information based on guidance for EFA (Henson & Roberts, 2006) and CFA (Jackson et al., 2009). As many previous studies have assessed other measurement properties of the EDE‐Q (e.g., construct reliability) and the current study looks in detail at structural validity, the full COSMIN risk of bias tool (and a potential 116 items) is not appropriate. For example, questions assessing the relevance of each questionnaire item or whether a comparator instrument was included were felt not to be pertinent and some COSMIN items covering methodological quality criteria differ from suggestions from EFA‐ or CFA‐specific guidance (e.g., sample size and missing data). In addition, although COSMIN guidance provides one section concerning structural validity (Mokkink et al., 2018), one of three relevant questions affords a higher score (and thus lower risk of bias) to studies which have included CFA as opposed to EFA. Given the aims of this study to appraise both EFA and CFA studies, it was decided to adapt COSMIN items on sample size and internal consistency. More detail is provided in Table S2 but, briefly, studies were accorded a score of either 1 or 0 for 10 items (seven each for EFA and CFA, and three across all studies) assessing elements of factor analysis reporting. A total score was therefore taken as an indicator of study quality. Where one paper reported both EFA and CFA, two separate scores were computed.

Studies were synthesized narratively and are presented according to the predominant sample characteristics. Meta‐analysis was considered but decided against due to the wide inclusion criteria (e.g., age, geography, and methods) which would have introduced significant “clinical” and “methodological” heterogeneity and potentially obscure genuine differences across samples (Deeks et al., 2021). Recognizing that reporting findings for different subgroups might be of interest, Table 1 presents study findings according to sample characteristics. Cohen's κ was computed for some key binary outcomes to estimate inter‐rater reliability of the coding scheme; two were assessed for EFA (Use of parallel analysis, κ = 1.00; Total variance reported, κ = 1.00) and two for CFA (Discussion of missing data, κ = .81; Cutoff criteria reported a priori, κ = .83).

## 3 RESULTS

### 3.1 Study selection

The results of the search and selection process are presented in Figure S1. After removing duplicates, 1410 papers were identified, of which 60 were included after screening full‐texts. Three studies of note were excluded from the systematic review—all because the full texts were published in languages other than English, and it was therefore not possible to make a full assessment of their methods (Gu et al., 2017; Hilbert et al., 2007; Pennings & Wojciechowski, 2004). One further study (Richter et al., 2018) was excluded for the same reason but seemed to offer a narrative review of measures rather than LVA of the EDE‐Q. The study of Mohd Taib and Khaiyom (2020) was included, although it was stated in the paper that this was a pilot study preceding another using different participants (Mohd Taib et al., 2021).

### 3.2 Study characteristics

Table 1 summarizes the study characteristics. A range of sample sizes were included in the LVA (range = 94–9910; mean = 1056; median = 565), from a total of 63,389 participants. Although Youth versions of the EDE‐Q were excluded from the search, several studies included individuals aged under 18, with an age range of included studies of 11–95 years. Body mass index (BMI) ranged from 12.23 to 107.9 kg/m^2^. Nineteen different language versions of the EDE‐Q were included, and samples were recruited from 26 different countries, although the modal country was the USA (one‐third of all studies; Table S3).

Best practice guidance typically recommends use of EFA to generate hypotheses about latent structures, which are subjected to CFA in different samples (e.g., Osborne, 2014). Studies of the EDE‐Q showed some evidence of this (with many of the most recent studies using CFA), although examples of EFA recur, often citing inconsistent findings regarding the EDE‐Q's latent structure as justification (e.g., Peterson et al., 2020). In total, there were 26 reports of EFA and 46 reports of CFA across the 60 studies.

The majority of studies included exclusively (*k* = 16; 27%) or predominantly (*k* = 53; 88%) female participants. Aside from the study of Peterson et al. (2020), which recruited a sample of transgender youth, there were three studies which explicitly stated genders other than male or female, with .1% (Rand‐Giovannetti et al., 2020), .25% (Jenkins & Davey, 2020), and 1.3% (Zickgraf et al., 2020) of the respective samples comprising other gender identities. Some were more evenly balanced across genders (e.g., Klimek et al., 2021), although predominantly non‐female samples tended to be purposively sampled (e.g., Peterson et al., 2020; Scharmer et al., 2020). Only a minority of studies (around one‐quarter) recruited from clinical settings, with nearly half of these including both clinical and non‐clinical participants.

### 3.3 Results of syntheses

#### 3.3.1 Latent structures

Where tested, studies using CFA failed to find support for the “original” four‐factor structure of the EDE‐Q, with two exceptions (Franko et al., 2012; Villarroel et al., 2011), although several caveats should be noted. Using a Spanish translation of the EDE‐Q in college women, Villarroel et al. (2011) reported “satisfactory” fit, noting that they “decided to assume the 4‐factor model proposed and theoretically justified by the original authors” (p. 124). Franko et al. (2012) used parceling as part of their analyses, which may lead to better fit than item‐indicator models, particularly on the fit indices used (Comparative Fit Index [CFI] and Root Mean Square Error of Approximation [RMSEA], the latter of which was relatively high at .12). Of 26 studies reporting EFA, nine (34.6%) generated a four‐factor solution although none perfectly replicated the Original model. In addition, four studies appeared to offer support for either a three‐ or four‐factor solution, depending on the criteria used to determine eligible factors, and one used a “forced” four‐factor solution in EFA which resulted in different interpretation from the Original.

The Weight Concern and Shape Concern subscales have been found to be highly correlated, and several studies in the current review generated latent structures through EFA whereby items of these subscales were considered under a “Weight and Shape Concern” subscale (e.g., Carey et al., 2019; Darcy et al., 2013; White et al., 2014). There was mixed evidence for the presence of a “Global” index of eating pathology, with several studies (Friborg et al., 2013; Klimek et al., 2021; Rand‐Giovannetti et al., 2020) generating conflicting findings regarding higher‐order models, suggesting that interpretation of the Global score might remain cautious, particularly in non‐female or ethnic minority groups (Goel, Burnette et al., 2022). Similarly, whilst some studies found that a one‐factor solution emerged from EFA (e.g., Peterson et al., 2020), others failed to find support using CFA (e.g., Calugi et al., 2017; Penelo et al., 2013). Investigations of the “full” (i.e., 22‐item) measure using CFA (Table S4) offered some support for a three‐factor model (Peterson et al., 2007) and a four‐factor model departing from the “Original” (Goel, Burnette et al., 2022; Friborg et al., 2013), although further work in different samples is needed. Interestingly, these models show similarities, such as combining Weight and Shape Concern into one factor (Rand‐Giovannetti et al., 2020).

Studies of briefer versions of the EDE‐Q tended to report positive results in terms of model fit, often suggesting preference over longer alternatives (e.g., Machado et al., 2020). The version proposed by Grilo et al. (2010), originally for the EDE but since applied to the EDE‐Q (e.g., Grilo et al., 2013), comprises seven items from the original EDE‐Q and provides three subscales: Dietary Restraint (three items); Shape/Weight Overvaluation (two items); and Body Dissatisfaction (two items). This model has been supported across several studies and samples (Table S6) and seems particularly well‐suited to assessing aspects of eating pathology in university student populations (Jenkins & Davey, 2020). A proposed alternative to this which has received some support is a one‐factor solution, developed in a sample of adolescent female twins by Wade et al. (2008), comprising eight items. However, the items within this are very similar to the “Weight and Shape Concern” subscales suggested by Friborg et al. (2013) and Peterson et al. (2007), suggesting that Weight and Shape Concern is a reliable construct that can be assessed through several items of the EDE‐Q.

### 3.4 Study quality and certainty of evidence

As noted above, studies were generally of moderate‐good quality (interquartile range for EFA = 4.25–8; for CFA = 6–8) and overall quality ratings suggested that many studies adequately reported a number of key elements of LVA. Those less frequently reported include the input matrix and communalities for EFA (46% and 15% of studies, respectively) and a relevant statement about normality and the matrix analyzed for CFA (57% and 26%).

Several studies included LVA as a secondary aim, often to establishing norms (e.g., Villarroel et al., 2011). However, there has been little replication of latent structures of the EDE‐Q, with some studies failing to find support with CFA and subsequently generating a novel version of the EDE‐Q using EFA.

## 4 DISCUSSION

The current review included 60 studies comprising over 60,000 participants, confirming that the EDE‐Q is a widely used self‐report measure for the assessment of eating pathology. The structural validity of the EDE‐Q has been investigated across a range of BMIs and ages, across five continents and 19 languages. Validation has included individuals with varying dietary choices (e.g., Heiss et al., 2020) and gender identities (e.g., Peterson et al., 2020), and both adolescents and adults have been studied, often in mixed samples. However, despite this wealth of research, the four‐factor solution commonly reported has not been consistently supported.

### 4.1 Structural validity of the EDE‐Q

The lack of support for the “Original” structure (Restraint, Eating Concern, Shape Concern, and Weight Concern) was perhaps unsurprising. The assertions of previous authors regarding flaws in the EDE‐Q seem, therefore, to be partially supported, although some of these “serious limitations” (Forbush et al., 2013, p. 861) may be driven by inconsistent interpretation of the factor structure of the “full” EDE‐Q. For example, the “linear dependency” between the Shape Concern and Weight Concern subscales (Parker et al., 2016, p. 567) suggests that they are measuring the same variable (or that there is little to discern worries about shape or weight), possibilities highlighted by the developers of the EDE (e.g., Cooper et al., 1989). This conclusion is supported by several studies in the current review endorsing aggregation of relevant items under a “combined” subscale (e.g., Barnes et al., 2012; Rand‐Giovannetti et al., 2020). There was limited evidence supporting the constructs of Restraint and Eating Concern (the latter of which was not included in the original conceptualization of the EDE‐Q; Fairburn & Beglin, 1994), with some studies suggesting removal and/or reclassification of these items (e.g., Parker et al., 2015; Penelo et al., 2013; White et al., 2014).

For full‐item models, strongest support appears to exist for those of Friborg et al. (2013) and Peterson et al. (2007) and, with briefer models, that of Grilo et al. (2010, 2013) has been investigated across several samples (Table S6). Given that many studies have made modifications to latent structures, it is difficult to say whether differences in factor structure are consistent across subgroups such as gender or diagnostic status, although some items of the EDE‐Q appear to lack measurement stability, particularly across groups (e.g., Compte et al., 2019; Rand‐Giovannetti et al., 2020).

Turning to the identification of a “Global” score, a bifactor (or “nested”) model, where a latent ‘Global’ factor reflecting common variance across all items is orthogonal (uncorrelated) to the EDE‐Q subscales, performed well compared to models with correlated subscales (Friborg et al., 2013), suggesting that the EDE‐Q Global score represents a useful measure of eating pathology and may thus be a valid indicator of treatment outcome (Tatham et al., 2015). However, given that few studies have explicitly addressed this issue, further work is required in light of other work challenging the computation of a “Global” score (Rand‐Giovannetti et al., 2020), perhaps through greater correspondence with other clinical indicators (Goel, Burnette et al., 2022).

More consistent support was found for a brief, seven‐item measure (the EDE‐Q7; Grilo et al., 2013), which seems to circumvent some of the issues with the longer measure (although admittedly sacrifices a degree of thoroughness). Interestingly, the EDE‐Q7 seems to demonstrate structural validity even when “behavioral” items (e.g., assessing binge eating) are included in LVA (e.g., Lev‐Ari et al., 2021) and some short versions combining behavioral and attitudinal items have resulted in adequate one‐factor solutions (e.g., He et al., 2021). Further studies might therefore look at the reliability and measurement invariance of brief versions comprising both attitudinal and behavioral items and establish whether the addition of behavioral items is necessary for the clinical utility of the EDE‐Q7.

### 4.2 Study quality

Looking at the quality of studies, sample sizes were often presented alongside justification and/or discussion and methodological details of EFA such as stating the estimation method and rotation and providing a pattern matrix. Of note, five EFA studies reported using principal component analysis (PCA), not EFA, which are conceptually (and mathematically) distinct procedures (Fabrigar et al., 1999) and several studies based factor extraction on the Kaiser–Guttman criterion (often referred to as the “Eigenvalues >1 Rule”), despite recommendations against this (Fabrigar et al., 1999; Henson & Roberts, 2006; Osborne, 2014). Thus, future research should continue to report important details of EFA procedures, use multiple criteria for factor extraction (e.g., Fabrigar et al., 1999; Henson & Roberts, 2006), and employ oblique rotation methods, given high inter‐item (e.g., Hilbert et al., 2012) and inter‐scale correlations.

Findings were similar in CFA studies, with issues such as internal consistency, normality, and discussion of missing data often mentioned. Reporting cutoffs for fit indices was common (but see Clark & Bowles, 2018), and, in general (e.g., Jackson et al., 2009), future studies should include both incremental and absolute measures of fit. Reporting of some indices (e.g., goodness‐of‐fit index [GFI]) are recommended against (Jackson et al., 2009, p. 10) and should perhaps be phased out.

Several studies generated novel latent structures using EFA, with few having subsequently been subject to rigorous evaluation through CFA. Several studies have set out to compare the performance of different models through CFA rather than generating additional novel solutions in future samples, particularly given the exploratory, and at times volatile, nature of EFA (Osborne, 2014). Such studies (e.g., Calugi et al., 2017; Goel, Burnette et al., 2022; Machado et al., 2020; Rand‐Giovannetti et al., 2020; Scharmer et al., 2020) are usually preferable to those evaluating the fit of only one model (Jackson et al., 2009) and, although more data are needed—particularly in under‐represented groups—findings appear to suggest (statistical) superiority of briefer models, particularly that attributed to Grilo et al., 2015) (Table S6). Whilst it should also be borne in mind that a “perfect” latent structure of the EDE‐Q may not emerge, further (confirmatory) validation of 22‐item (e.g., Friborg et al., 2013) and brief versions (e.g., Gideon et al., 2016; Grilo et al., 2013) seems warranted, as well as greater investigation into the optimal construction of a “Global” score.

### 4.3 Recommendations for use of the EDE‐Q in clinical and research settings

As has been previously argued (e.g., Friborg et al., 2013), reliance on the “Original” (four‐factor) interpretation of EDE‐Q scores should be avoided unless there is a strong rationale for doing otherwise. If the full scale is being used, it would seem wise to report Weight and Shape Concern items as a composite measure or, at least, to conduct appropriate sensitivity analyses (such as a combined Weight and Shape Concern subscale, or by deriving subscales of the EDE‐Q7) (e.g., Hilbert et al., 2020; Mason et al., 2018). We recommend that users should consider how best to employ the EDE‐Q (or related measures) in light of their aims. The 22‐item EDE‐Q may be appropriate in certain cases—perhaps using the Global score as a measure of outcome—but the interpretation of scores based on the “original” subscales seems to lack justification in terms of structural validity. Further work is required, however, to be sure that the 22‐item Global score can be usefully compared between different populations, such as men and women, and greater scrutiny of the clinical utility of different versions of the EDE‐Q is recommended.

Given the availability of a brief version (EDE‐Q7; Grilo et al., 2010) and apparently strong support for its psychometric structure and invariance across samples (e.g., Machado et al., 2018; Rand‐Giovannetti et al., 2020), wider implementation in clinical (and research) settings seems warranted, particularly where clinicians and researchers might be concerned about item and scale performance. Given that the EDE‐Q7 has received support across several samples and the measure can be meaningfully derived from the full version, this might provide an appropriate assessment of ED psychopathology—specifically, the constructs of dietary restraint, body dissatisfaction, and overvaluation of weight and shape. In addition, inclusion of both behavioral and attitudinal items is possible when the scales (scoring) are adapted (e.g., Gideon et al., 2016; He et al., 2021) and may offer a helpful compromise between comprehensiveness of symptom assessment and psychometric validity.

To further evaluate longer versions of the EDE‐Q, it would seem worthwhile to use techniques such as multidimensional item response theory, in combination with those from “classical test theory” (e.g., He et al., 2021), to better determine the precision and reliability of individual items (Osteen, 2010), and to assess the performance of a “Global” score in predicting treatment outcome, for example. Further comparison of alternative versions, particularly in clinical groups, seems warranted, in addition to critical evaluation of the predictive validity of subscales and investigation in more diverse samples.

### 4.4 Recommendations for reporting of factor analyses

Whilst the overall quality of reporting was good, the matrix (for both EFA and CFA) was not usually specified, although it could be inferred in some cases (e.g., through reference to specific software). This finding is common in methodology reviews of factor analysis as many statistics programmes have this as a default, but should nonetheless be stated explicitly (Jackson et al., 2009). Researchers should continue to report key elements of factor analysis methods (mindful of the influence of “default” program settings) and also note matrices and estimation methods wherever possible. Consistent with the recommendations of methodologists, we encourage researchers to consider their aims and choose appropriate strategies for employing factor analysis to ensure that the results are both generalisable and interpretable (Osborne, 2014; Preacher et al., 2013). Finally, given the ordinal nature of “attitudinal” items on the EDE‐Q, appropriate robust estimation methods should be used in CFA (e.g., Rhemtulla et al., 2012).

### 4.5 Limitations

There were some limitations of this review which bear mention. Behavioral items were typically excluded from factor analyses, and hence this review—partly as the EDE‐Q suggests a ratio (rather than ordinal) scoring for these items. Future work might consider how these items can be integrated into a consistent scoring framework (e.g., Forbush et al., 2013; He et al., 2021). Detailed discussion of structural validity was limited to EFA and CFA, although some alternatives (e.g., Rasch analysis) were identified in the searches and are noted (e.g., Gideon et al., 2016; He et al., 2021). Three articles were found through reviewing reference lists which, although a minority of those included in the final review (5.0%), were not identified through database searching.

Although the latent structure of the EDE‐Q has been investigated in many countries, none from the continent of Africa was identified. Studies of EDs in (particularly Southern) Africa since the 1970s suggest that their presence is more complex than simple “Westernization” and requires greater cultural understanding (Szabo & Le Grange, 2001), indicating that replication attempts in African samples would be informative. Perhaps surprisingly, relatively few studies have included exclusively clinical samples, with some including these as part of a larger sample for LVA (e.g., Machado et al., 2014). As a result, the factor structure of the EDE‐Q in clinical samples remains under‐studied. Information on participants' socioeconomic status was reported in 25% (15/60) of included studies, usually according to different criteria (e.g., parents' highest education, household income).

Although most studies (*k* = 44, 73.3%) included information on race or ethnicity, sample characteristics were sometimes unclear and, despite the wealth of studies, there remains a need for future research on samples with greater diversity, particularly regarding gender and ethnicity, to enhance generalizability to historically under‐represented groups (Goel, Jennings Mathis, et al., 2022). In line with reporting in treatment trials (Burnette et al., 2022), data on race/ethnicity were often focused on “White,” often including a binary distinction between “White” and “Other.” Moving forward, studies should collect (and report) detailed data rather than broad categories (Burnette et al., 2022), and provide data on all represented races/ethnicities, not just the majority group. Papers not in English were excluded from the review and one highly cited paper in particular (Hilbert et al., 2007) may have been helpful to include as it seems to have influenced several subsequent empirical studies. Lastly, translated versions of the EDE‐Q were included and it is possible that this may have influenced the findings, for example, due to errors in translation (Hawkins et al., 2020).

## 5 CONCLUSIONS

This systematic review of 60 studies offers evidence that reporting of subscale scores according to the originally proposed factor structure of the EDE‐Q is not supported in the peer‐reviewed literature (Thomas et al., 2014). The EDE‐Q7 (Grilo et al., 2010, 2013) offers promise and can perhaps combine the intent and relevance of the “original” EDE‐Q with a more psychometrically robust factor structure. Further research looking at the clinical utility of the EDE‐Q7 would be valuable, as well as greater scrutiny of “youth” versions of the EDE‐Q and whether adjustments are needed for younger samples.

## AUTHOR CONTRIBUTIONS

**Paul Jenkins:** Conceptualization; data curation; formal analysis; methodology; writing – original draft; writing – review and editing. **Renee Rienecke:** Conceptualization; data curation; formal analysis; methodology; writing – original draft; writing – review and editing.

## Supporting information


**Appendix S1** Supporting Information

## Data Availability

The study protocol has been published. As a systematic literature review, data were extracted from existing papers and tabulated and synthesized (see Tables and Supplemental Material). The quality appraisal tool used is described in the manuscript and Supplemental Material.
