# Supplementary material for: Structural validity of the Eating Disorder Examination—Questionnaire: A systematic review
Source: Int J Eat Disord. 2022 May 3;55(8):1012–30. doi: 10.1002/eat.23721 (PMC9543786; doi:10.1002/eat.23721)
Supplement: Supplementary file 1 — Appendix S1 Supporting Information [file EAT-55-1012-s001.docx]

Table S1. PRISMA Checklist

| **Section and Topic** | **Item #** | **Checklist item** | **Location where item is reported** |
| --- | --- | --- | --- |
| **TITLE** | | |  |
| Title | 1 | Identify the report as a systematic review. | Title |
| **ABSTRACT** | | |  |
| Abstract | 2 | See the PRISMA 2020 for Abstracts checklist. | Abstract |
| **INTRODUCTION** | | |  |
| Rationale | 3 | Describe the rationale for the review in the context of existing knowledge. | Introduction |
| Objectives | 4 | Provide an explicit statement of the objective(s) or question(s) the review addresses. | Introduction |
| **METHODS** | | |  |
| Eligibility criteria | 5 | Specify the inclusion and exclusion criteria for the review and how studies were grouped for the syntheses. | Protocol and registration |
| Information sources | 6 | Specify all databases, registers, websites, organisations, reference lists and other sources searched or consulted to identify studies. Specify the date when each source was last searched or consulted. | Search strategy |
| Search strategy | 7 | Present the full search strategies for all databases, registers and websites, including any filters and limits used. | Search strategy |
| Selection process | 8 | Specify the methods used to decide whether a study met the inclusion criteria of the review, including how many reviewers screened each record and each report retrieved, whether they worked independently, and if applicable, details of automation tools used in the process. | Data extraction |
| Data collection process | 9 | Specify the methods used to collect data from reports, including how many reviewers collected data from each report, whether they worked independently, any processes for obtaining or confirming data from study investigators, and if applicable, details of automation tools used in the process. | Data extraction |
| Data items | 10a | List and define all outcomes for which data were sought. Specify whether all results that were compatible with each outcome domain in each study were sought (e.g., for all measures, time points, analyses), and if not, the methods used to decide which results to collect. | Data extraction |
|  | 10b | List and define all other variables for which data were sought (e.g., participant and intervention characteristics, funding sources). Describe any assumptions made about any missing or unclear information. | Data extraction |
| Study risk of bias assessment | 11 | Specify the methods used to assess risk of bias in the included studies, including details of the tool(s) used, how many reviewers assessed each study and whether they worked independently, and if applicable, details of automation tools used in the process. | Quality assessment and data synthesis |
| Effect measures | 12 | Specify for each outcome the effect measure(s) (e.g., risk ratio, mean difference) used in the synthesis or presentation of results. | N/A |
| Synthesis methods | 13a | Describe the processes used to decide which studies were eligible for each synthesis (e.g., tabulating the study intervention characteristics and comparing against the planned groups for each synthesis (item #5)). | Quality assessment and data synthesis |
|  | 13b | Describe any methods required to prepare the data for presentation or synthesis, such as handling of missing summary statistics, or data conversions. | Quality assessment and data synthesis |
|  | 13c | Describe any methods used to tabulate or visually display results of individual studies and syntheses. | Quality assessment and data synthesis |
|  | 13d | Describe any methods used to synthesize results and provide a rationale for the choice(s). If meta-analysis was performed, describe the model(s), method(s) to identify the presence and extent of statistical heterogeneity, and software package(s) used. | Quality assessment and data synthesis |
|  | 13e | Describe any methods used to explore possible causes of heterogeneity among study results (e.g., subgroup analysis, meta-regression). | N/A |
|  | 13f | Describe any sensitivity analyses conducted to assess robustness of the synthesized results. | N/A |
| Reporting bias assessment | 14 | Describe any methods used to assess risk of bias due to missing results in a synthesis (arising from reporting biases). | Data synthesis, Supplementary Material |
| Certainty assessment | 15 | Describe any methods used to assess certainty (or confidence) in the body of evidence for an outcome. | N/A |
| **RESULTS** | | |  |
| Study selection | 16a | Describe the results of the search and selection process, from the number of records identified in the search to the number of studies included in the review, ideally using a flow diagram. | Figure S1 |
|  | 16b | Cite studies that might appear to meet the inclusion criteria, but which were excluded, and explain why they were excluded. | Study selection,  Figure S1 |
| Study characteristics | 17 | Cite each included study and present its characteristics. | Table 1 |
| Risk of bias in studies | 18 | Present assessments of risk of bias for each included study. | Supplementary Material |
| Results of individual studies | 19 | For all outcomes, present, for each study: (a) summary statistics for each group (where appropriate) and (b) an effect estimate and its precision (e.g. confidence/credible interval), ideally using structured tables or plots. | N/A |
| Results of syntheses | 20a | For each synthesis, briefly summarise the characteristics and risk of bias among contributing studies. | Supplementary Material,  Tables S3 and S4 |
|  | 20b | Present results of all statistical syntheses conducted. If meta-analysis was done, present for each the summary estimate and its precision (e.g. confidence/credible interval) and measures of statistical heterogeneity. If comparing groups, describe the direction of the effect. | N/A |
|  | 20c | Present results of all investigations of possible causes of heterogeneity among study results. | N/A |
|  | 20d | Present results of all sensitivity analyses conducted to assess the robustness of the synthesized results. | N/A |
| Reporting biases | 21 | Present assessments of risk of bias due to missing results (arising from reporting biases) for each synthesis assessed. | Study quality and certainty of evidence,  Supplementary Material,  Tables S3 and S4 |
| Certainty of evidence | 22 | Present assessments of certainty (or confidence) in the body of evidence for each outcome assessed. | Study quality and certainty of evidence |
| **DISCUSSION** | | |  |
| Discussion | 23a | Provide a general interpretation of the results in the context of other evidence. | Discussion |
|  | 23b | Discuss any limitations of the evidence included in the review. | Discussion |
|  | 23c | Discuss any limitations of the review processes used. | Limitations |
|  | 23d | Discuss implications of the results for practice, policy, and future research. | Recommendations |
| **OTHER INFORMATION** | | |  |
| Registration and protocol | 24a | Provide registration information for the review, including register name and registration number, or state that the review was not registered. | Protocol & Registration |
|  | 24b | Indicate where the review protocol can be accessed, or state that a protocol was not prepared. | Protocol & Registration |
|  | 24c | Describe and explain any amendments to information provided at registration or in the protocol. | N/A |
| Support | 25 | Describe sources of financial or non-financial support for the review, and the role of the funders or sponsors in the review. | Role of funding source |
| Competing interests | 26 | Declare any competing interests of review authors. | Conflict of interest |
| Availability of data, code and other materials | 27 | Report which of the following are publicly available and where they can be found: template data collection forms; data extracted from included studies; data used for all analyses; analytic code; any other materials used in the review. | N/A |

*From:*  Page MJ, McKenzie JE, Bossuyt PM, Boutron I, Hoffmann TC, Mulrow CD, et al. The PRISMA 2020 statement: an updated guideline for reporting systematic reviews. BMJ 2021;372:n71. doi: 10.1136/bmj.n71

For more information, visit: <http://www.prisma-statement.org/>

**Risk of bias assessment**

Included studies are assessed using a 10-item checklist. This includes an item (*Internal consistency*) based on COSMIN guidance (Mokkink et al., 2018), used across all studies. In addition, two items relevant to both EFA and CFA were included across all studies (*Sample size* [also mentioned in COSMIN guidance], *Software used*). Fourteen separate items specific to either EFA (Henson & Roberts, 2006) or CFA (Jackson et al., 2009) are included. To assess risk of bias for each study, items are afforded a score of 1 when reporting is deemed complete or 0 if data is missing or not reported completely. The total score for an EFA or CFA study is therefore 10. Each reviewer provided a score independently, and any differences were resolved through discussion and re-assessment of the original study. Of note, a Cochrane item on “Incomplete outcome data” was considered as part of Item 12 for CFA (‘Handing Missing Data’).

Table S2. Risk of bias item descriptions

| Item | Label and description | Support for inclusion | Scoring |
| --- | --- | --- | --- |
| **All studies** | | |  |
| 1 | Sample size: Does the study report this, with specific reference to the latent variable analysis (LVA)? | Sample size can affect variability and precision of estimates of population loadings  (Note: this criterion was based on the authors reporting a *justification* of sample size. Scoring was not based on a view of whether the sample size was appropriate for LVA – in part due to inconsistent guidance regarding this – but, rather, whether readers were given sufficient information to relate the sample size to LVA.) | If reference to / justification of sample size given, score 1 |
| 2 | Software used: Does the study report the computer software used? | Different software can have different methods of estimating fit / extracting variables | If software reported, score 1 |
| 3 | Internal consistency: Was a statement / discussion about internal consistency mentioned? | Estimates the degree of interrelatedness among items (Mokkink et al., 2018, p. 11) | If statement about internal consistency present, score 1 |
| **EFA (Henson & Roberts, 2006)** | | |  |
| 4 | Input matrix: Does the study specify the matrix used to compare relationships between variables? | The default tends to be correlation matrix (p. 397), although difference input matrices may not result in the same fit of a measurement model | If input matrix reported, score 1 |
| 5 | Estimation method: Does the study report the method used to remove common variance from the input matrix? | Different methods can have an influence on interpretation | If estimation method reported, score 1 |
| 6 | Rotation: Does the study report the method of rotation? | Rotation strategies are numerous and can determine whether factors are hypothesised to correlate to one another (‘oblique’) or are orthogonal | If rotation method reported, score 1 |
| 7 | Factor retention rules: Was a suitable approach taken to determine the number of factors to retain? | Different ‘rules’ can lead to different decisions regarding the number of factors to retain (p. 399). Parallel analysis is often favoured, in addition to use of multiple criteria (Fabrigar et al., 1999) | If parallel analysis used, score 1. If multiple methods (e.g., scree test + Kaiser-Guttman criterion), score 1. |
| 8 | Number of factors: Does the study clearly report the number of latent factors identified in EFA? | An essential outcome, which can affect construct validity of scores (p. 403) | If number of factors clearly reported, score 1 |
| 9 | Pattern matrix: Is a pattern matrix reported in the main paper (or directed elsewhere, e.g., Supporting Material)? | Full reporting affords “independent interpretation of the final results” (p. 400). Although reporting of all item-factor correlations is recommended (e.g., Schreiber, 2021), we considered this present if a partial pattern matrix was presented (e.g., if suppressed loadings were not reported). | If pattern matrix reported, score 1 |
| 10 | Communalities: Are communalities (shared variance) reported? | Enhances external evaluation (p. 408) | If communalities reported, score 1 |
| **CFA (Jackson et al., 2009)** | | |  |
| 11 | Normality: Does the study provide information on (non-)normality of the data? | Non-normal data can lead to overestimations of the chi-square statistic | If statement about data normality present, score 1 |
| 12 | Handling missing data: Does the study report information on how missing data was handled (e.g., multiple imputation)? | Presence of missing data can bias fit parameter estimates or increase the likelihood of convergence failures, even when missing at random (p. 9) | If statement about missing data present, score 1 |
| 13 | Estimation method: Does the study specify the method used for estimating model parameters? | This, too, can be affected by normality and sample size | If estimation method reported, score 1 |
| 14 | Input matrix: Does the study specify the matrix used to compare measured variables to those implied by a hypothesised model? | The default tends to be the variance-covariance matrix, although some authors analyse the correlation matrix, which may necessitate scale invariance (p. 10) | If input matrix method reported, score 1 |
| 15 | Fit indices: Which fit indices were used in the study? | Many indices have different properties, and some have been recommended against (e.g., GFI, AGFI, NFI). A balance of different measures (e.g., an incremental fit index and a residuals-based index) is recommended (p. 10) | If at least 2 used (including one incremental index and one absolute index), score 1 |
| 16 | Cutoff criteria: Were cutoffs for fit indices reported a priori? | Despite some controversy, it is recommended that any cutoffs used are stated clearly and a priori (p. 12) | If *a priori* cutoff criteria reported, score 1 |
| 18 | Models tested: How many models were tested / compared? | It is recommended that CFA should compare two or more models that are “theoretically plausible” (p. 19) | If more than one model compared, score 1 |

Figure S1. PRISMA flow diagram showing the selection of studies of the EDE-Q’s factor structure (adapted from Page et al., 2021)

**Identification of studies via other methods**

**Identification of studies via databases and registers**

Records removed *before screening*:

Duplicate records removed

(n = 89)

Records identified from:

Websites (n = 0)

Citation searching (n = 4)

Records identified from

Databases (n = 1497)

Eating disorders listserv (n = 2)

**Identification**

Records screened

(n = 4)

Records excluded

(n = 1332)

Records screened

(n = 1410)

Reports not retrieved

(n = 4 [Full text not in English])

Reports sought for retrieval

(n = 4)

Reports sought for retrieval

(n = 78)

**Screening**

Reports excluded (n = 17):

Not a factor analysis of the EDE-Q (n = 3)

Combined the EDE-Q with another scale (n = 3)

Youth version of EDE-Q (n = 4)

Did not use all items of EDE-Q, i.e., only subscales (n = 2)

EDE (Interview) not EDE-Q (n = 2)

Not the EDE-Q (a novel measure) (n = 1)

Corrigendum (n = 1)

In the process of peer-review (n = 1)

Reports excluded:

Not a factor analysis of the EDE-Q (n = 1)

Reports assessed for eligibility

(n = 4)

Reports assessed for eligibility

(n = 74)

Studies included in review

(n = 60)

**Included**

Table S3. Summary of Exploratory Factor Analysis elements reported in studies, including quality assessment rating

| First author, year | Soft-ware | Input Matrix | Estimation Method | Rotation | Scree plot reported | Total variance reported | Eigenvalue Rule | Parallel analysis | Number of factors | Pattern matrix reported | Communality reported | Quality score |
| --- | --- | --- | --- | --- | --- | --- | --- | --- | --- | --- | --- | --- |
| Aardoom, 2012 | SPSS | NR | PCA | Oblimin | No | Yes | Yes | No | 4 | Yes | No | 6 |
| Becker, 2010 | NR | Corr | PAF | Promax | No | Yes | No | No | 4 – forced | Yes | No | 6 |
| Carey, 2019 | SPSS | Corr | PAF | Promax | No | Yes | Yes | Yes | 3 | Yes | No | 8 |
| Darcy, 2013 | SPSS | NR | ML | Promax | No | Yes | No | Yes | 3 | Yes | No | 7 |
| Forbush, 2011 | SAS | NR | NR | Promax | No | Yes | No | Yes | 3 – forced | Yes | No | 7 |
| Friborg, 2013 | SPSS | Corr | PAF | Promax | No | Yes | Yes | Yes | 3, 4^a^ | Yes | Yes | 8 |
| Gideon, 2016 | SPSS | NR | PCA | Oblimin | Yes | Yes | Yes | No | 5 | Yes | No | 7 |
| Hilbert, 2012 | SPSS | NR | PCA | Varimax | Yes | Yes | Yes | No | 3 | Yes | Yes | 8 |
| Hrabosky, 2008 | NR | Corr | PAF | Oblimin | No | Yes | Yes | No | 4 | Yes | No | 6 |
| Lewis-Smith, 2021 | Mplus 8 | Corr | WLSMV | Geomin | Yes | No | Yes | Yes | 2 | Yes | No | 8 |
| Machado, 2014 | NR | NR | NR | Oblimin | No | Yes | No | No | 3 | Yes | No | 4 |
| Melisse, 2021 | SPSS | NR | ML | Promax | Yes | Yes | No | No | 3, 4^a^ | No | No | 4 |
| Mitsui, 2017 | SPSS | NR | PCA | Equamax | Yes | Yes | Yes | No | 4 | Yes | No | 7 |
| Mohd Taib, 2020 | SPSS | NR | NR | NR | No | No | Yes | No | 6 | No | No | 7 |
| Mohd Taib, 2021 | SPSS | NR | PAF | Oblimin | Yes | Yes | Yes | No | 4 | Yes | No | 3 |
| Otani, 2021 | SPSS | NR | UWLS | Equamax | Yes | Yes | No | No | 3 | No | No | 5 |
| Parker, 2015 | SPSS | Corr | ML | Varimax | Yes | Yes | Yes | Yes | 4 | Yes | No | 9 |
| Parker, 2016 | SPSS | Corr | PAF | Oblimin | Yes | Yes | Yes | Yes | 4 | No | No | 7 |
| Peterson, 2007 | NR | NR | PAA^b^ | Promax | No | Yes | No | No | 3, 4^a^ | Yes | No | 4 |
| Peterson, 2020 | Mplus | Corr | WLSMV | Geomin | Yes | Yes | Yes | Yes | 1 | Yes | Yes | 9 |
| Phillips, 2018 | SPSS | Corr | PAF | Promax | Yes | Yes | Yes | No | 4 | Yes | No | 8 |
| Prnjak, 2020 | R | Corr | PAF | NR | No | Yes | No | Yes | 4 | Yes | Yes | 8 |
| Ramli, 2008 | NR | NR | NR | Varimax | No | Yes | Yes | No | 4 | Yes | No | 4 |
| White, 2014 | SPSS | Corr | PAF | Promax | Yes | Yes | Yes | No | 3 | Yes | No | 8 |
| Wood, 2016 | NR | NR | PCA | Varimax | No | Yes | Yes | No | 5 | Yes | No | 4 |
| Zohar, 2017 | SPSS | NR | NR | Varimax | No | Yes | No | No | 3, 4^a^ | Yes | No | 4 |

*Note*. ^a^These studies produced more than one proposed factor solution (e.g., using different ‘rules’ for factor extraction). ^b^PAA = Principal axis analysis, but this was likely PAF; Corr = Correlation; ML = Maximum likelihood; NR = Not reported; PAF = Principal axis factoring; PCA = Principal components analysis; UWLS = Unbiased weighted least square; WLSMV = Weighted least squares with means and variances adjusted.

Table S4. Summary of Confirmatory Factor Analysis elements reported in studies, including quality assessment rating

| First author, year | Normality statement | Discussion of missing data | Estimation procedure | Software | Matrix analyzed | Fit indices used | Cutoff criteria reported a priori | Models tested | Internal consistency reported | Quality score |
| --- | --- | --- | --- | --- | --- | --- | --- | --- | --- | --- |
| Allen, 2011 | No | No | NR | Lisrel | Corr | GFI, AGFI, NFI, CFI, RMSEA | Yes | 5 | Yes | 6 |
| Asl, 2021 | Yes | Yes | ML | Lisrel | NR | CFI, NFI, NNFI, SRMR, RMSEA, IFI, GFI, RFI, AGFI | Yes | 1 | Yes | 8 |
| Baceviciene, 2020 | Yes | Yes | NR | Amos | NR | CFI, TLI, GFI, AGFI, RMSEA | Yes | 3 | Yes | 7 |
| Barnes, 2012 | No | Yes | ML, Robust ML | Amos, EQS | NR | CFI, NFI, chi-square, RMSEA | No | 3 | Yes | 6 |
| Calugi, 2017 | No | Yes | Robust ML | Mplus | NR | CFI, TLI, RMSEA, SRMR | Yes | 3 | Yes | 7 |
| Carey, 2019 | Yes | Yes | Bootstrapped ML | Amos | Cov | GFI, NFI, CFI, AGFI, RMSEA, chi-square | Yes | 6 | Yes | 10 |
| Carrard, 2015 | No | No | NR | Mplus | NR | CFI, RMSEA, SRMR | Yes | 3 | Yes | 5 |
| Chan, 2015 | No | No | NR | Amos | NR | CFI, IFI, NFI, TLI, RMSEA | Yes | 5 | Yes | 5 |
| Compte, 2019 | Yes | Yes | Robust ML | R | NR | CFI, TLI, RMSEA, SRMR, chi-square | Yes | 5 | Yes | 8 |
| Darcy, 2013 | Yes | Yes | Robust ML | Lisrel | NR | Satorra-Bentler scaled chi-square, CFI, TLI, SRMR, modification indices | Yes | 1 | No | 7 |
| Franko, 2012 | No | No | NR | Amos | NR | GFI, CFI, RMSEA | No | 1 | Yes | 3 |
| Friborg, 2013 | Yes | No | NR | Lisrel | Corr | TLI, RMSEA, AIC, BIC | Yes | 5 | Yes | 7 |
| Giovazolias, 2013 | Yes | No | ULS | Amos | Cov | GFI, AGFI, NFI, SRMR, AIC | Yes | 4 | Yes | 8 |
| Goel, 2022 | Yes | Yes | WLSMV | R lavaan | NR | CFI, TLI, RMSEA, SRMR, ECVI | Yes | 12 | Yes | 9 |
| Grilo, 2013 | No | Yes | ML | Mplus | NR | CFI, TLI, RMSEA, SRMR | Yes | 2 | Yes | 8 |
| Grilo, 2015 | No | Yes | ML | Mplus | NR | CFI, TLI, RMSEA, SRMR | Yes | 3 | Yes | 7 |
| He, 2021 | Yes | No | WLSMV | R | NR | CFI, TLI, RMSEA | Yes | 1 | Yes | 6 |
| Heiss, 2018 | Yes | Yes | FIML | SAS | NR | CFI, GFI, AGFI, RMSEA, TLI, SRMR | No | 5 | Yes | 7 |
| Heiss, 2020 | Yes | Yes | NR | SAS | NR | CFI, GFI, AGFI, RMSEA, TLI, SRMR | Yes | 1 | Yes | 8 |
| Hrabosky, 2008 | No | No | NR | Lisrel | NR | CFI, RMSEA, SRMR | Yes | 1 | Yes | 4 |
| Jenkins, 2020 | Yes | Yes | FIML | Amos | NR | CFI, TLI, RMSEA, SRMR, chi-square | Yes | 1 | Yes | 8 |
| Kliem, 2016 | No | Yes | Robust ML | R | NR | CFI, TLI, RMSEA, SRMR | Yes | 2 | Yes | 7 |
| Klimek, 2021 | Yes | Yes | WLSMV | R | NR | CFI, RMSEA, SRMR | Yes | 3 | Yes | 9 |
| Levi-Ari, 2021 | No | No | NR | Amos | Corr | CFI, RMSEA, SRMR | Yes | 1 | Yes | 4 |
| Lewis-Smith, 2021 | No | Yes | NR | MPlus | Corr | CFI, TLI, RMSEA, SRMR, chi-square | Yes | 1 | Yes | 6 |
| Lichtenstein, 2021 | Yes | No | Non-parametric bootstrap sampling | Stata | NR | CFI, TLI, RMSEA, SRMR | No | 1 | Yes | 5 |
| McEntee, 2021 | No | Yes | ML | Mplus | Cov | CFI, TLI, RMSEA, SRMR, chi-square, AIC | Yes | 5 | Yes | 9 |
| Machado, 2018 | No | Yes | ML | Mplus | NR | CFI, TLI, RMSEA | Yes | 2 | Yes | 7 |
| Machado, 2020 | No | Yes | WLSMV | Mplus | NR | CFI, TLI, RMSEA, SRMR, pClose | Yes | 4 | Yes | 7 |
| Melisse, 2021 | Yes | Yes | NR | Amos | NR | CFI, TLI, GFI, AGFI, NFI, IFI, RMSEA | No | 6 | Yes | 6 |
| Otani, 2021 | No | No | NR | Amos | NR | CFI, RMSEA | No | 2 | Yes | 4 |
| Parker, 2015 | Yes | Yes | ML with bootstrap-  ping | Amos and Mplus | Cov | CFI, RMSEA, chi-square | Yes | 1 | Yes | 9 |
| Parker, 2016 | Yes | Yes | ML with bootstrap-ping | Amos and Mplus | Cov | CFI, RMSEA, chi-square | Yes | 2 | Yes | 9 |
| Penelo, 2013 | Yes | Yes | Robust ML | Mplus | Cov | CFI, RMSEA, chi-square | No | 4 | Yes | 9 |
| Prnjak, 2020 | Yes | Yes | NR | R | NR | CFI, TLI, RMSEA, SRMR | No | 5 | Yes | 6 |
| Rand-Giovannetti, 2020 | No | Yes | ULSMV | Mplus | NR | CFI, TLI, RMSEA | Yes | 21 | No | 6 |
| Rica, 2021 | Yes | Yes | ULSMV | Mplus | NR | CFI, TLI, RMSEA | Yes | 4 | Yes | 8 |
| Scharmer, 2020 | Yes | Yes | Robust ML | Mplus | NR | CFI, TLI, RMSEA, SRMR | Yes | 7 | Yes | 9 |
| Serier, 2018 | Yes | No | WLSMV | Mplus | Cov | CFI, TLI, RMSEA, WRMR | Yes | 6 | Yes | 9 |
| Serier, 2021 | Yes | Yes | WLSMV | Mplus | NR | CFI, TLI, RMSEA, SRMR | Yes | 1 | Yes | 7 |
| Tobin, 2019 | Yes | Yes | Robust ML | Mplus | NR | CFI, TLI, RMSEA | Yes | 2 | Yes | 8 |
| Unikel Santoncini, 2018 | No | No | ML | Stata | NR | CFI, TLI, RMSEA, SRMR | Yes | 3 | Yes | 6 |
| Villarroel, 2011 | Yes | Yes | Robust ML | Lisrel | NR | CFI, TLI, RMSEA, SRMR | No | 1 | Yes | 6 |
| White, 2014 | Yes | Yes | ML with bootstrap-  ping | Amos | Cov | CFI, NFI, GFI, AGFI, RMSEA | Yes | 1 | Yes | 9 |
| Zickgraf, 2020 | No | No | WLSMV | Mplus | NR | CFI, RMSEA, SRMR | Yes | 1 | Yes | 6 |
| Zohar, 2017 | No | No | ML | Amos | NR | CFI, TLI, RMSEA, SRMR | No | 2 | Yes | 5 |

*Note*. AGFI = Adjusted goodness of fit index; AIC = Akaike’s Information Criterion; BIC = Bayesian information criterion; CFI = Comparative fit index; Corr = Correlation; Cov = Covariance; ECVI = Expected Cross-Validation Index; GFI = Goodness of fit index; IFI = Incremental fit index; ML = Maximum likelihood; NFI = Normed fit index; NR = Not reported; pClose = p of Close Fit; RFI = Relative fit index; RMSEA = Root mean square error of approximation; SRMR = Standardized root mean square residual; TLI = Tucker-Lewis index (also known as the non-normed fit index, or NNFI); ULSMV = Mean-and variance-adjusted unweighted least squares; WLSMV = Mean- and variance-adjusted weighted least squares; WRMR = Weighted root mean square residual

Table S5. EDE-Q language versions identified in the review

| Language | Number of studies |
| --- | --- |
| Arabic | 1 |
| Chinese | 2 |
| Croatian | 1 |
| Danish | 1 |
| Dutch | 1 |
| English | 30 |
| Fijian | 1 |
| French | 1 |
| German | 2 |
| Greek | 1 |
| Hebrew | 2 |
| Italian | 1 |
| Japanese | 2 |
| Lithuanian | 1 |
| Malay | 3 |
| Norwegian | 1 |
| Persian | 1 |
| Portuguese | 3 |
| Spanish (including Mexican-Spanish) | 5 |

| First author and model | Number of studies supporting model | Studies supporting model |
| --- | --- | --- |
| **Full models** | | |
| Peterson et al.’s (2007) 3-factor model | 2 | Barnes et al. (2012); Giovazolias et al. (2013) |
| Fairburn et al.’s (2008) 4-factor model | 2 | Franko et al. (2012); Villarroel et al. (2011) |
| Friborg et al.’s (2013) 4-factor model | 3 | Friborg et al. (2013); Klimek et al. (2021);  Rand-Giovannetti et al. (2020) |
| **Reduced-item models** | | |
| Wade et al.’s (2008) 1-factor model | 3 | Allen et al. (2011); Chan et al. (2015); Compte et al. (2019) |
| Darcy et al.’s (2013) 3-factor model (female nonathlete) | 2 | Darcy et al. (2013); Rand-Giovannetti et al. (2020) |
| Darcy et al.’s (2013) 3-factor model  (male athlete) | 2 | Darcy et al. (2013); Rand-Giovannetti et al. (2020) |
| Grilo et al.’s (2010) 3-factor model | 16 | Calugi et al. (2017); Carrard et al. (2015); Grilo et al. (2013); Grilo et al. (2015); Heiss et al. (2020); Jenkins et al. (2020); Klimek et al. (2021); Machado et al. (2018); Machado et al. (2020); McEntee et al. (2021);  Rand-Giovannetti et al. (2020); Scharmer et al. (2020); Serier et al. (2019); Serier et al. (2021); Tobin et al. (2019); Unikel Santoncini et al. (2018); Zickgraf et al. (2020) |

Table S6. EDE-Q models receiving support from at least two studies using confirmatory factor analysis

References to articles solely included in the systematic review

Aardoom, J. J., Dingemans, A. E., Slof Op’t Landt, M. C. T., & van Furth, E. F. (2012). Norms and discriminative validity of the Eating Disorder Examination Questionnaire (EDE-Q). *Eating Behaviors, 13*, 305-309. https://doi.org/10.1016/j.eatbeh.2012.09.002

Allen, K. L., Byrne, S. M., Lampard, A., Watson, H., & Fursland, A. (2011). Confirmatory factor analysis of the Eating Disorder Examination-Questionnaire (EDE-Q). *Eating Behaviors, 12*, 143-151. https://doi.org/10.1016/j.eatbeh.2011.01.005

Asl, E. M., Mahaki, B., Khanjani, S., & Mohammadian, Y. (2021). Assessment of eating disorder psychopathology: The psychometric properties of the Persian version of the Eating Disorder Examination Questionnaire Short Form. Journal of Research in Medical Sciences, 26(1), 71. https://doi.org/10.4103/jrms.jrms_230_20

Baceviciene, M., Balciuniene, V., & Jankauskiene, R. (2020). Validation of the Lithuanian version of the Eating Disorder Examination Questionnaire 6.0 in a student sample. *Brain and Behavior, 10*, e01555. https://doi.org/10.1002/brb3.1555

Carrard, I., Rebetez, M. M. L., Mobbs, O., & Van der Linden, M. (2015). Factor structure of a French version of the eating disorder examination-questionnaire among women with and without binge eating disorder symptoms. *Eating and Weight Disorders, 20*, 137-144. https://doi.org/10.1007/s40519-014-0148-x

Chan, C. W., & Leung, S. F. (2015). Validation of the Eating Disorder Examination Questionnaire: An online version. *Journal of Human Nutrition and Dietetics, 28*, 659-665. https://doi.org/10.1111/jhn.12275

Forbush, K. T. (2011). *Examining the structure of eating pathology through scale construction*. [Doctoral dissertation, University of Iowa]. ProQuest LLC

Giovazolias, T., Tsaousis, I., & Vallianatou, C. (2013). The factor structure and psychometric properties of the Greek version of the eating disorders examination questionnaire (EDE-Q). *European Journal of Psychological Assessment, 29*, 189-196. https://psycnet.apa.org/doi/10.1027/1015-5759/a000138

Kliem, S., Mößle, T., Zenger, M., Strauß, B., Brähler, E., & Hilbert, A. (2016). The eating disorder examination-questionnaire 8: A brief measure of eating disorder psychopathology (EDE-Q8). *International Journal of Eating Disorders, 49*, 613-616. https://doi.org/10.1002/eat.22487

Lewis-Smith, H., Garbett, K. M., Chaudhry, A., Uglik-Marucha, N., Vitoratou, S., Dhillon, M., Shroff, H., & Diedrichs, P. C. (2021). Adaptation and validation of the Eating Disorder Examination-Questionnaire in English among urban Indian adolescents. *International Journal of Eating Disorders, 54*, 187-202. https://doi.org/10.1002/eat.23431

Lichtenstein, M. B., Haastrup, L., Johansen, K. K., Bindzus, J. B., Larsen, P. V., Støving, R. K., Clausen, L., & Linnet, J. (2021). Validation of the Eating Disorder Examination Questionnaire in Danish eating disorder patients and athletes. *Journal of Clinical Medicine, 10*(17), 3976. https://doi.org/10.3390/jcm10173976

Machado, P. P. P., Grilo, C. M., & Crosby, R. D. (2018). Replication of a modified factor structure for the Eating Disorder Examination-Questionnaire: Extension to clinical eating disorder and non-clinical samples in Portugal. *European Eating Disorders Review, 26*, 75-80. https://doi.org/10.1002/erv.2569

McEntee, M. L., Serier, K. N., Smith, J. M., & Smith, J. E. (2021). The sum is greater than its parts: Intersectionality and measurement validity of the Eating Disorder Examination Questionnaire (EDE-Q) in Latinx undergraduates in the United States. *Sex Roles, 84*, 102-111. https://doi.org/10.1007/s11199-020-01149-7

Melisse, B., van Furth, E. F., & de Beurs, E. (2021). Eating disorder examination questionnaire (EDE-Q): validity and norms for Saudi nationals. *Eating and Weight Disorders*. https://doi.org/10.1007/s40519-021-01150-3.

Otani, M., Hiraide, M., Horie, T., Mitsui, T., Yoshida, T., Takamiya, S., Sakuta, R., Usami, M., Komaki, G., & Yoshiuchi, K. (2021). Psychometric properties of the Eating Disorder Examination-Questionnaire and psychopathology in Japanese patients with eating disorders. *International Journal of Eating Disorders, 54*, 203-211. https://doi.org/10.1002/eat.23452

Phillips, K. E., Jennings, K. M., & Gregas, M. (2018). Factor structure of the eating disorder examination-questionnaire in a clinical sample of adult women with anorexia nervosa. *Journal of Psychosocial Nursing and Mental Health Services, 56*, 33-39. https://doi.org/10.3928/02793695-20180108-03

Prnjak, K., & Jukic, I. (2020). Development and validation of the Croatian version of Eating Disorder Examination Questionnaire in a community sample. *Eating and Weight Disorders, 26*, 859-868. https://doi.org/10.1007/s40519-020-00915-6

Ramli, M., Jamaiyah, H., Noor, A., Khairani, O., & Adam, B. (2008). Cross-cultural adaptation and validation of the Bahasa Malaysia version of the Eating Disorder Examination Questionnaire (EDE-Q). *Malaysian Journal of Psychiatry, 17*.

Rica, R., Solar, M., Compte, E. J., & Sepúlveda, A. R. (2021). Establishing the optimal male cut-off point: confirmatory factor analysis of the eating disorder examination-questionnaire (EDE-Q) in a representative sample of Spanish university students. *Eating and Weight Disorders*. https://doi.org/10.1007/s40519-021-01234-0

Serier, K. N., Smith, J. E., & Yeater, E. A. (2018). Confirmatory factor analysis and measurement invariance of the Eating Disorder Examination Questionnaire (EDE-Q) in a non-clinical sample of non-Hispanic White and Hispanic women. *Eating Behaviors, 31*, 53-59. https://doi.org/10.1016/j.eatbeh.2018.08.004

Serier, K. N., Peterson, K. P., VanderJagt, H., Sebastian, R. M., Mullins, C. R., Medici, J., Smith, J. M., & Smith, J. E. (2021). Factor analytic support for the EDE-Q7 among American Indian/Alaska Native undergraduate women. *Eating and Weight Disorders*. https://doi.org/10.1007/s40519-021-01335-w

Tobin, L. N., Lacroix, E., & von Ranson, K. M. (2019). Evaluating an abbreviated three-factor version of the Eating Disorder Examination Questionnaire in three samples. *Eating Behaviors, 32*, 18-22. https://doi.org/10.1016/j.eatbeh.2018.11.003

Unikel Santoncini, C., Bojorquez Chapela, I., Díaz de León Vázquez, C., Vázquez Velázquez, V., Rivera Márquez, J. A., Galván Sánchez, G., & Rocha Velis, I. (2018). Validation of eating disorders examination questionnaire in Mexican women. *International Journal of Eating Disorders, 51*, 146-154. https://doi.org/10.1002/eat.22819

Wood, A. M. (2016). *Underlying Processes in the Development of Eating Disorder Symptoms*. [Doctoral dissertation, Roosevelt University]. ProQuest LLC

Zohar, A. H., Lev-Ari, L., & Bachner-Melman, R. (2017). The EDE-Q in Hebrew: Structural and convergent/divergent validity in a population sample. *Israel Journal of Psychiatry, 54*, 15-21.
